# *catena*-Poly[4-methyl­morpholin-4-ium [[dichloridobismuth(III)]-di-μ-chlorido]]

**DOI:** 10.1107/S1600536812001717

**Published:** 2012-01-21

**Authors:** Ying-Chun Wang

**Affiliations:** a College of Chemistry and Chemical Engineering, Southeast University, Nanjing 210096, People’s Republic of China

## Abstract

The asymmetric unit of the title complex, {(C_5_H_12_NO)[BiCl_4_]}_*n*_, contains two bridging and two *cis* non-bridging chloride ligands coordinated to a central Bi^III^ atom, and one 4-methyl­morpholin-4-ium cation. The Bi^III^ atoms are linked by the bridging chloride ligands into linear chains parallel to the *c* axis. The chloride ions create a pseudo-octa­hedral geometry about each Bi^III^ atom. Bifurcated N—H⋯Cl hydrogen bonds link the cations to the anionic chains.

## Related literature

For the structures of related amino compounds, see: Turnbull (2007 ▶). For the ferroelectric properties of related amino derivatives, see: Fu *et al.* (2011*a* ▶,*b* ▶,*c* ▶). 
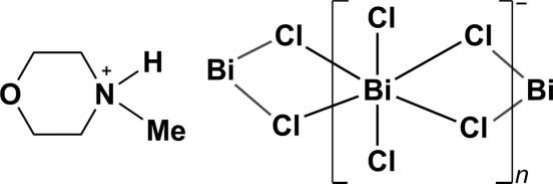


## Experimental

### 

#### Crystal data

(C_5_H_12_NO)[BiCl_4_]
*M* *_r_* = 452.94
Monoclinic, 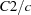

*a* = 18.166 (4) Å
*b* = 9.801 (2) Å
*c* = 13.915 (3) Å
β = 93.36 (3)°
*V* = 2473.2 (9) Å^3^
*Z* = 8
Mo *K*α radiation
μ = 15.08 mm^−1^
*T* = 298 K
0.10 × 0.05 × 0.05 mm

#### Data collection

Rigaku Mercury2 diffractometer
Absorption correction: multi-scan (*CrystalClear*; Rigaku, 2005 ▶) *T*_min_ = 0.428, *T*_max_ = 0.470
12468 measured reflections
2830 independent reflections
2437 reflections with *I* > 2σ(*I*)
*R*_int_ = 0.090

#### Refinement

*R*[*F* ^2^ > 2σ(*F* ^2^)] = 0.037
*wR*(*F* ^2^) = 0.088
*S* = 1.14
2830 reflections
111 parameters
H-atom parameters constrained
Δρ_max_ = 2.45 e Å^−3^
Δρ_min_ = −1.44 e Å^−3^

### 

Data collection: *CrystalClear* (Rigaku, 2005 ▶); cell refinement: *CrystalClear*; data reduction: *CrystalClear*; program(s) used to solve structure: *SHELXS97* (Sheldrick, 2008 ▶); program(s) used to refine structure: *SHELXL97* (Sheldrick, 2008 ▶); molecular graphics: *SHELXTL* (Sheldrick, 2008 ▶); software used to prepare material for publication: *SHELXTL*.

## Supplementary Material

Crystal structure: contains datablock(s) I, global. DOI: 10.1107/S1600536812001717/pk2380sup1.cif

Structure factors: contains datablock(s) I. DOI: 10.1107/S1600536812001717/pk2380Isup2.hkl

Additional supplementary materials:  crystallographic information; 3D view; checkCIF report

## Figures and Tables

**Table 1:** Hydrogen-bond geometry (Å, °)

*D*—H⋯*A*	*D*—H	H⋯*A*	*D*⋯*A*	*D*—H⋯*A*
N1—H1⋯Cl3^i^	0.90	2.76	3.434 (6)	133
N1—H1⋯Cl2^i^	0.90	2.85	3.410 (6)	122

Symmetry code: (i) 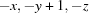.

**Fig. 1.**
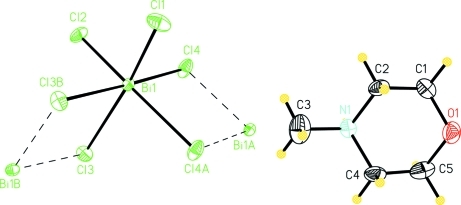
A view of the title compound. Displacement ellipsoids are drawn at the 30% probability level. H atoms are represented as small spheres of arbitrary radii.

**Fig. 2.**
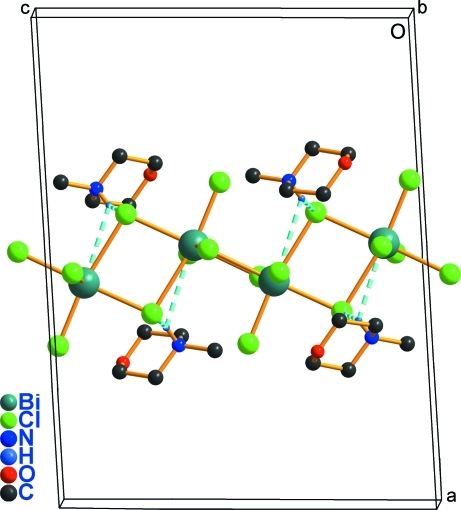
The crystal packing of the title compound showing the one-dimensional chain. Hydrogen atoms not involved in hydrogen bonding have been omitted for clarity.

## supplementary crystallographic information

### Comment

Simple organic salts containing amino cations have attracted attention as materials that display ferroelectric-paraelectric phase transitions (Fu *et al.*, 2011*a*,*b*,*c*). In this study, we describe the crystal structure of the title compound, *N*-methylmorpholinium *catena*-Poly[(di-*µ_2_-*chloro)-dichloro Bi^III^]

The asymmetric unit contains four independent Cl atoms, one Bi^III^ atom and one organic cation (Fig. 1). All bond lengths and angles are normal and comparable with those reported for the cation in a related Ni(III) compound (Turnbull, 2007). The non-bridging (Cl1 & Cl2) and bridging Cl (Cl3 & Cl4) atoms create a pseudo-octahedral geometry about each Bi (III) atom. The Bi^III^ atoms are linked *via* bridging Cl ions into linear chains that propagate parallel to the *c* axis.

In the crystal structure, the amino N1 atom is involved in hydrogen bonds with the Cl atoms (Cl2 and Cl3) with the N—H···Cl distance of 3.434 (6) and 3.410 (6) Å, respectively. The bifurcated N—H···Cl H-bonds link the cations to the inorganic anion chain. (Fig. 2, Table 1).

### Experimental

A mixture of N-methylmorpholine (0.4 mmol), BiCl_3_ (0.4 mmol) and HCl/distilled water (10ml, 1:4) sealed in a Teflon-lined stainless steel vessel was maintained at 100 °C. Colorless block crystals suitable for X-ray analysis were obtained after 3 days.

### Refinement

All H atoms attached to C atoms were fixed geometrically and treated as riding with C-H = 0.97 Å (methylene) and C-H = 0.96 Å (methyl) with *U*_iso_(H) = 1.2*U*_eq_ (methylene) and *U*_iso_(H) = 1.5*U*_eq_ (methyl). Positional parameters of the N-bound H atom were intially refined freely, but subsequently restrained using a distance of 0.90 Å and, in the final refinements treated as riding on their parent nitrogen atoms with *U_iso_*(H)=1.2*U_eq_*(N).

### Crystal data

**Table:**

(C_5_H_12_NO)[BiCl_4_]	*F*(000) = 1664
*M**_r_* = 452.94	*D*_x_ = 2.433 Mg m^−^^3^
Monoclinic, *C*2/*c*	Mo *K*α radiation, λ = 0.71073 Å
Hall symbol: -C 2yc	Cell parameters from 2830 reflections
*a* = 18.166 (4) Å	θ = 3.6–27.5°
*b* = 9.801 (2) Å	µ = 15.08 mm^−^^1^
*c* = 13.915 (3) Å	*T* = 298 K
β = 93.36 (3)°	Block, colorless
*V* = 2473.2 (9) Å^3^	0.10 × 0.05 × 0.05 mm
*Z* = 8	

### Data collection

**Table:**

Rigaku Mercury2 diffractometer	2830 independent reflections
Radiation source: fine-focus sealed tube	2437 reflections with *I* > 2σ(*I*)
graphite	*R*_int_ = 0.090
Detector resolution: 13.7 pixels mm^-1^	θ_max_ = 27.5°, θ_min_ = 3.6°
CCD profile fitting scans	*h* = −23→23
Absorption correction: multi-scan (*CrystalClear*; Rigaku, 2005)	*k* = −12→12
*T*_min_ = 0.428, *T*_max_ = 0.470	*l* = −18→18
12468 measured reflections	

### Refinement

**Table:**

Refinement on *F*^2^	Secondary atom site location: difference Fourier map
Least-squares matrix: full	Hydrogen site location: inferred from neighbouring sites
*R*[*F*^2^ > 2σ(*F*^2^)] = 0.037	H-atom parameters constrained
*wR*(*F*^2^) = 0.088	*w* = 1/[σ^2^(*F*_o_^2^) + (0.015*P*)^2^ + 5.1391*P*] where *P* = (*F*_o_^2^ + 2*F*_c_^2^)/3
*S* = 1.14	(Δ/σ)_max_ < 0.001
2830 reflections	Δρ_max_ = 2.45 e Å^−^^3^
111 parameters	Δρ_min_ = −1.44 e Å^−^^3^
0 restraints	Extinction correction: *SHELXL97* (Sheldrick, 2008), Fc^*^=kFc[1+0.001xFc^2^λ^3^/sin(2θ)]^-1/4^
Primary atom site location: structure-invariant direct methods	Extinction coefficient: 0.0095 (2)

### Special details

**Table:**

Geometry. All esds (except the esd in the dihedral angle between two l.s. planes) are estimated using the full covariance matrix. The cell esds are taken into account individually in the estimation of esds in distances, angles and torsion angles; correlations between esds in cell parameters are only used when they are defined by crystal symmetry. An approximate (isotropic) treatment of cell esds is used for estimating esds involving l.s. planes.
Refinement. Refinement of F^2^ against ALL reflections. The weighted R-factor wR and goodness of fit S are based on F^2^, conventional R-factors R are based on F, with F set to zero for negative F^2^. The threshold expression of F^2^ > 2sigma(F^2^) is used only for calculating R-factors(gt) etc. and is not relevant to the choice of reflections for refinement. R-factors based on F^2^ are statistically about twice as large as those based on F, and R- factors based on ALL data will be even larger.

### Fractional atomic coordinates and isotropic or equivalent isotropic displacement parameters (Å^2^)

**Table:**

	*x*	*y*	*z*	*U*_iso_*/*U*_eq_	
Bi1	0.038523 (12)	0.36337 (2)	−0.101352 (17)	0.02581 (15)	
Cl4	−0.02461 (14)	0.3417 (2)	0.06880 (17)	0.0494 (6)	
Cl3	−0.10115 (11)	0.3718 (2)	−0.22296 (15)	0.0407 (5)	
Cl2	0.02635 (11)	0.1057 (2)	−0.11236 (16)	0.0438 (5)	
Cl1	0.16693 (12)	0.3571 (2)	−0.02381 (18)	0.0568 (7)	
N1	0.1587 (3)	0.9444 (6)	0.1521 (4)	0.0342 (14)	
H1	0.1239	0.9003	0.1835	0.041*	
O1	0.1918 (3)	1.1380 (6)	0.3004 (5)	0.0564 (18)	
C2	0.2251 (4)	0.9334 (8)	0.2192 (6)	0.045 (2)	
H2A	0.2371	0.8380	0.2299	0.054*	
H2B	0.2667	0.9766	0.1907	0.054*	
C1	0.2122 (4)	0.9995 (9)	0.3123 (5)	0.046 (2)	
H1B	0.1735	0.9510	0.3432	0.055*	
H1C	0.2568	0.9938	0.3540	0.055*	
C4	0.1376 (5)	1.0896 (9)	0.1428 (6)	0.049 (2)	
H4A	0.1754	1.1389	0.1106	0.058*	
H4B	0.0917	1.0974	0.1040	0.058*	
C5	0.1287 (5)	1.1502 (9)	0.2384 (8)	0.059 (3)	
H5A	0.1166	1.2461	0.2305	0.070*	
H5B	0.0878	1.1060	0.2675	0.070*	
C3	0.1707 (6)	0.8803 (10)	0.0586 (8)	0.075 (3)	
H3A	0.1258	0.8837	0.0185	0.113*	
H3B	0.1852	0.7869	0.0685	0.113*	
H3C	0.2088	0.9284	0.0277	0.113*	

### Atomic displacement parameters (Å^2^)

**Table:**

	*U*^11^	*U*^22^	*U*^33^	*U*^12^	*U*^13^	*U*^23^
Bi1	0.0243 (2)	0.0229 (2)	0.0303 (2)	0.00046 (9)	0.00217 (12)	−0.00330 (11)
Cl4	0.0746 (15)	0.0284 (11)	0.0480 (12)	−0.0059 (9)	0.0276 (11)	−0.0025 (9)
Cl3	0.0254 (10)	0.0589 (15)	0.0384 (11)	0.0057 (7)	0.0067 (8)	0.0025 (9)
Cl2	0.0409 (11)	0.0212 (10)	0.0692 (15)	0.0001 (8)	0.0033 (10)	−0.0040 (10)
Cl1	0.0340 (12)	0.0697 (18)	0.0647 (15)	−0.0136 (9)	−0.0135 (10)	0.0200 (11)
N1	0.035 (3)	0.036 (4)	0.033 (3)	−0.012 (3)	0.010 (2)	0.003 (3)
O1	0.052 (4)	0.049 (4)	0.068 (4)	−0.010 (3)	−0.003 (3)	−0.010 (3)
C2	0.029 (4)	0.033 (5)	0.071 (6)	0.001 (3)	−0.002 (4)	0.006 (4)
C1	0.040 (5)	0.058 (6)	0.040 (5)	−0.011 (4)	0.002 (3)	0.007 (4)
C4	0.050 (5)	0.037 (5)	0.058 (6)	−0.003 (4)	−0.004 (4)	0.021 (5)
C5	0.043 (6)	0.046 (6)	0.088 (8)	0.005 (4)	0.009 (5)	0.018 (5)
C3	0.080 (8)	0.070 (8)	0.078 (8)	−0.035 (5)	0.018 (6)	−0.012 (6)

### Geometric parameters (Å, °)

**Table:**

Bi1—Cl1	2.513 (2)	C2—C1	1.480 (11)
Bi1—Cl2	2.539 (2)	C2—H2A	0.9700
Bi1—Cl4	2.699 (2)	C2—H2B	0.9700
Bi1—Cl3^i^	2.758 (2)	C1—H1B	0.9700
Bi1—Cl4^ii^	2.939 (2)	C1—H1C	0.9700
Bi1—Cl3	2.967 (2)	C4—C5	1.475 (13)
Cl4—Bi1^ii^	2.939 (2)	C4—H4A	0.9700
Cl3—Bi1^i^	2.758 (2)	C4—H4B	0.9700
N1—C3	1.473 (12)	C5—H5A	0.9700
N1—C4	1.476 (9)	C5—H5B	0.9700
N1—C2	1.486 (9)	C3—H3A	0.9600
N1—H1	0.9000	C3—H3B	0.9600
O1—C5	1.399 (11)	C3—H3C	0.9600
O1—C1	1.415 (10)		
			
Cl1—Bi1—Cl2	94.42 (7)	C1—C2—H2B	109.5
Cl1—Bi1—Cl4	93.03 (9)	N1—C2—H2B	109.5
Cl2—Bi1—Cl4	86.25 (6)	H2A—C2—H2B	108.1
Cl1—Bi1—Cl3^i^	87.74 (8)	O1—C1—C2	111.8 (7)
Cl2—Bi1—Cl3^i^	90.88 (6)	O1—C1—H1B	109.3
Cl4—Bi1—Cl3^i^	177.08 (6)	C2—C1—H1B	109.3
Cl1—Bi1—Cl4^ii^	92.51 (7)	O1—C1—H1C	109.3
Cl2—Bi1—Cl4^ii^	168.41 (7)	C2—C1—H1C	109.3
Cl4—Bi1—Cl4^ii^	84.11 (6)	H1B—C1—H1C	107.9
Cl3^i^—Bi1—Cl4^ii^	98.68 (6)	C5—C4—N1	110.5 (7)
Cl1—Bi1—Cl3	170.68 (7)	C5—C4—H4A	109.5
Cl2—Bi1—Cl3	85.68 (6)	N1—C4—H4A	109.5
Cl4—Bi1—Cl3	96.28 (7)	C5—C4—H4B	109.5
Cl3^i^—Bi1—Cl3	82.94 (6)	N1—C4—H4B	109.5
Cl4^ii^—Bi1—Cl3	88.98 (6)	H4A—C4—H4B	108.1
Bi1—Cl4—Bi1^ii^	95.89 (6)	O1—C5—C4	113.1 (7)
Bi1^i^—Cl3—Bi1	96.97 (6)	O1—C5—H5A	109.0
C3—N1—C4	112.6 (7)	C4—C5—H5A	109.0
C3—N1—C2	111.5 (7)	O1—C5—H5B	109.0
C4—N1—C2	108.8 (6)	C4—C5—H5B	109.0
C3—N1—H1	111.6	H5A—C5—H5B	107.8
C4—N1—H1	108.7	N1—C3—H3A	109.5
C2—N1—H1	103.1	N1—C3—H3B	109.5
C5—O1—C1	110.7 (6)	H3A—C3—H3B	109.5
C1—C2—N1	110.7 (6)	N1—C3—H3C	109.5
C1—C2—H2A	109.5	H3A—C3—H3C	109.5
N1—C2—H2A	109.5	H3B—C3—H3C	109.5

Symmetry codes: (i) −*x*, *y*, −*z*−1/2; (ii) −*x*, −*y*+1, −*z*.

### Hydrogen-bond geometry (Å, °)

**Table:**

*D*—H···*A*	*D*—H	H···*A*	*D*···*A*	*D*—H···*A*
N1—H1···Cl3^ii^	0.90	2.76	3.434 (6)	133
N1—H1···Cl2^ii^	0.90	2.85	3.410 (6)	122

Symmetry codes: (ii) −*x*, −*y*+1, −*z*.
